# FAD-YOLO: a lightweight feature-refined and task-aligned framework for AIS–MIA discrimination on pulmonary CT

**DOI:** 10.3389/fmed.2026.1881220

**Published:** 2026-06-25

**Authors:** Jinghui Chen, Tao Yang, Zhipeng Sun, Chengbin Ye, Lianxin Xie, Hongjia Zhao

**Affiliations:** 1 The First Clinical Medical College, The Affiliated People’s Hospital of Fujian University of Traditional Chinese Medicine, Fuzhou, Fujian, China; 2 The Affiliated People’s Hospital of Fujian University of Traditional Chinese Medicine, Fuzhou, Fujian, China

**Keywords:** adenocarcinoma *in situ*, ground-glass nodule, lung adenocarcinoma, minimally invasive adenocarcinoma, object detection, YOLO12

## Abstract

**Introduction:**

Adenocarcinoma *in situ* (AIS) and minimally invasive adenocarcinoma (MIA) typically present as ground-glass nodules (GGNs) on pulmonary computed tomography (CT), and their low contrast, blurred boundaries, and morphological variability pose substantial challenges to automated detection.

**Methods:**

Using YOLO12n as the baseline, we propose FAD-YOLO (Feature-refinement, Alignment, and Dynamic-sampling YOLO), a lightweight yet accurate detection framework with three task-oriented improvements: an A2C2f-FRFN module that replaces the standard feed-forward network within the Area-Attention C2f module with a Feature Refinement Feed-forward Network; a DySample dynamic upsampling module that replaces nearest-neighbor interpolation with content-aware learnable offsets; and a TADDH task-aligned dynamic detection head that combines a shared group-normalized convolution, deformable sampling in the localization branch, and layer-attention reweighting to alleviate classification–localization misalignment.

**Results:**

On an independent internal test set of 317 images, FAD-YOLO achieved a precision of 93.8%, a recall of 93.4%, an mAP@50 of 93.6%, and an mAP@50–95 of 70.8%, while reducing parameters by 18.7% relative to the baseline at the cost of a 12.5% increase in GFLOPs (from 6.4 to 7.2). Under identical settings, it performed comparably to or better than YOLOv5n, YOLOv8n, YOLO11n, and RT-DETR-R18, and outperformed the much larger RT-DETR-R50 on all accuracy metrics despite using approximately one-twentieth of its parameters. On an external Mendeley Data test set, the model achieved mAP@50 = 91.7% and mAP@50–95 = 68.2% without any fine-tuning.

**Discussion:**

FAD-YOLO achieves a favorable balance among accuracy, lightweight design, and cross-dataset generalization, and may serve as a candidate for further prospective validation as an aid to radiologists in AIS/MIA discrimination on resource-constrained clinical devices.

## 1 Introduction

Lung cancer ranks first in cancer-related mortality worldwide, with approximately 2.48 million new cases and 1.80 million deaths in 2022 (1); lung adenocarcinoma is the most common histological subtype, accounting for approximately 45% of non-small-cell lung cancers (2). According to the classification jointly issued by Travis et al. (3) and the International Association for the Study of Lung Cancer, preinvasive and early invasive lesions are subdivided into adenocarcinoma *in situ* (AIS) and minimally invasive adenocarcinoma (MIA). Both entities are defined by a maximum nodule diameter of no more than 3 cm; AIS shows purely lepidic growth without stromal invasion, whereas MIA shows predominantly lepidic growth with an invasive component of no more than 5 mm. The 5th edition of the WHO classification of thoracic tumors (4) retains these criteria, and the AIS/MIA definitions adopted here are consistent across both classifications. The meta-analysis by Behera et al. (5) reported a five-year disease-free survival rate approaching 100% after complete resection for both entities. Accurate early imaging-based discrimination between AIS and MIA therefore carries substantial clinical value for surgical planning, avoidance of overtreatment, and outcome improvement.

Computed tomography (CT) has become the central modality for lung disease screening and diagnosis owing to its high spatial resolution, rapid acquisition, and clear depiction of the pulmonary parenchyma (6, 7), with thin-section CT in particular providing detailed visualization of nodule density, morphology, and surrounding spatial relationships (8). Nevertheless, automated AIS/MIA discrimination on CT remains challenging. Wu et al. (9) noted that both lesions present as ground-glass opacities or ground-glass-predominant mixed nodules and are difficult to differentiate even for experienced radiologists; Gong et al. (10) further showed that ground-glass nodules (GGNs) exhibit substantial inter-class overlap in density, size, and morphology, while their blurred boundaries and low contrast, compounded by interference from pulmonary vessels, airways, and respiratory motion, render discrimination based solely on morphological features highly difficult. Efficient and reliable automated detection algorithms are therefore of pressing clinical importance.

Data-driven deep learning has progressively replaced traditional handcrafted-feature approaches, and convolutional neural networks (CNNs) have driven substantial advances in pulmonary nodule detection and classification (11, 12). Setio et al. (13) proposed Multi-View ConvNets on LUNA16, fusing nine fixed-orientation 2D slices to reduce false positives, but the method still depends on conventional CADe candidate generation and underuses true 3D information. Nibali et al. (14) employed ResNet-based transfer learning on LIDC-IDRI for benign-versus-malignant classification with a high area under the curve (AUC), although the model operates only on pre-cropped patches and is not end-to-end. Dou et al. (15) designed a Multilevel Contextual 3D CNN for false-positive reduction with leading competition performance metric (CPM) scores, at the cost of high memory and latency from three parallel 3D branches. For GGN invasiveness assessment, Sun et al. (16) fused radiomic and CNN features for invasiveness prediction yet still relied heavily on handcrafted features; Zhao et al. (17) developed a 3D fully convolutional network for three-class classification of AIS, MIA, and invasive adenocarcinoma but required extensive parameters and voxel-level annotations; and Lin et al. (18) incorporated a feature pyramid into Faster R-CNN to enhance GGN detection, although recall for sub-centimeter nodules remained sensitive to anchor settings. Overall, mainstream CNN detectors typified by Faster R-CNN suffer from heavy reliance on large annotated datasets, limited sensitivity to small GGNs, and laborious anchor tuning, all of which impede end-to-end lightweight deployment.

To overcome the limited receptive fields and weak long-range modeling of CNNs, Transformer-based methods have been introduced for pulmonary CT analysis. Ni et al. (19) proposed a 3D Attention Network that outperformed pure CNN baselines on a multi-center cohort, although full 3D self-attention substantially increases memory and training cost with input resolution. Ding et al. (20) developed a multi-task Transformer that jointly predicts the micropapillary subtype and invasiveness of lung adenocarcinoma with improved accuracy, but its parameter scale and FLOPs preclude real-time inference on bedside or portable devices. Gai et al. (21) systematically compared Swin Transformer, ViT, and mainstream CNNs on multi-center data, confirming the advantage of attention mechanisms for fine-grained feature characterization while highlighting the prohibitive quadratic complexity of global self-attention on high-resolution 3D CT, which limits real-time clinical deployment.

The need to balance computational efficiency with global modeling has driven the widespread adoption of one-stage real-time detectors, exemplified by the YOLO series, in medical imaging. Through successive iterations from YOLOv5 to YOLOv8 and YOLO11, the series has continually advanced in lightweight design and inference speed and has become a mainstream backbone for clinical and industrial deployment. The YOLO12 model proposed by Tian et al. (22) elevates attention from an auxiliary role to a core backbone component, achieving Transformer-comparable long-range modeling while preserving real-time inference through the A2C2f module, and YOLO12n exhibits a clear accuracy advantage over conventional purely convolutional YOLO variants, making it a strong candidate for resource-constrained medical imaging. However, YOLO12 is optimized for natural images and lacks dedicated medical-imaging adaptation. Litjens et al. (23) have noted that the direct transfer of general-purpose vision architectures to medical imaging inevitably encounters domain shift and intra-class heterogeneity, which is particularly pronounced in fine-grained AIS/MIA discrimination.

Three specific domain-adaptation limitations of YOLO12n on this task are identified. First, the standard feed-forward network within A2C2f performs only channel-wise transformations through two pointwise convolutions, lacks explicit modeling of local spatial details, and provides no mechanism to selectively emphasize discriminative activations or suppress redundant responses, thereby constraining its capacity to characterize the subtle density differences and fine-grained edge cues (e.g., spiculation, lobulation) critical in low-contrast GGNs. Second, the nearest-neighbor upsampling in the FPN-PAN neck is a static, content-agnostic operation that blurs boundary transition zones and internal density gradients, with particularly adverse effects on small AIS lesions with diffuse boundaries. Third, the decoupled detection head extracts spatial features independently in its classification and regression branches, producing spatial inconsistency between high-confidence classification regions and accurately localized bounding boxes and yielding redundant predictions for morphologically variable, blurred-boundary GGNs. Together, these limitations cap the achievable performance of YOLO12n.

To address these limitations, this paper proposes FAD-YOLO (Feature-refinement, Alignment, and Dynamic-sampling YOLO), in which three existing modules are adapted and systematically integrated to confront the typical challenges of AIS/MIA discrimination, namely low contrast, blurred boundaries, and morphological variability. Rather than introducing new architectural primitives, the principal contribution of this work lies in the task-oriented adaptation and systematic integration of existing components, as detailed below:

- An A2C2f-FRFN feature refinement module is formed by integrating FRFN into A2C2f, replacing the standard FFN. Through a gated, content-adaptive refinement, this module strengthens the characterization of subtle density differences and discriminative edge cues in GGNs.
- A DySample dynamic upsampling module is introduced to replace fixed nearest-neighbor interpolation with content-aware learnable offset sampling, preserving GGN boundary sharpness and density-transition continuity during upsampling.
- A TADDH task-aligned dynamic detection head is adopted, in which a shared group-normalized convolution extracts joint features for the two tasks, deformable convolution is applied within the localization branch to align sampling with lesion morphology, and layer-attention mechanisms perform task-specific reweighting, jointly aligning classification confidence with localization accuracy and effectively suppressing redundant predictions in AIS/MIA discrimination

## 2 YOLO12

As introduced above, YOLO12 (2025) makes attention the core of the backbone while preserving real-time inference. Unlike the purely convolutional aggregation modules in YOLOv8 and YOLO11 (e.g., C2f and C3k2), it introduces an Area Attention mechanism and combines it with the CSP structure to form the A2C2f (Area-Attention C2f) module. Area Attention partitions the feature map along the spatial dimension into several local regions and computes attention within each region, achieving approximately global modeling at linear complexity and thereby alleviating the quadratic-complexity bottleneck of standard self-attention.

Architecturally, YOLO12 keeps the standard three-stage backbone–neck–head design, with stacked A2C2f modules in the backbone, a bidirectional FPN–PAN neck using nearest-neighbor upsampling, and a decoupled head trained with Distribution Focal Loss and Task Alignment Learning. The three elements most relevant to this work—the A2C2f backbone module, the nearest-neighbor upsampling in the neck, and the decoupled head—are precisely the targets of the modifications introduced in Section 3.

For AIS/MIA discrimination on pulmonary CT, YOLO12 offers several task-relevant advantages: Area Attention within A2C2f captures global density distributions of GGNs against complex pulmonary parenchymal backgrounds without compromising real-time performance; the end-to-end one-stage prediction avoids the region-proposal dependency of two-stage detectors such as Faster R-CNN, reducing deployment complexity; and the five model variants (n/s/m/l/x) enable flexible adaptation to different computational budgets. These properties make YOLO12 better suited to this task than conventional purely convolutional YOLO variants.

Within this trend, the YOLO family has been increasingly adapted to pulmonary nodule detection: attention and upsampling-enhanced YOLOv8 variants have been proposed to improve sensitivity to small nodules (24), YOLO detectors have been combined with lung segmentation and Transformer modules to reduce false positives (25), and transfer-learning-based YOLOv7 pipelines have been applied to lung-cancer detection (26). These studies, however, largely address generic nodule localization or benign-versus-malignant screening; by contrast, FAD-YOLO targets the fine-grained, low-contrast AIS-versus-MIA discrimination of GGNs under a strict lightweight, real-time constraint.

**Figure 1:**
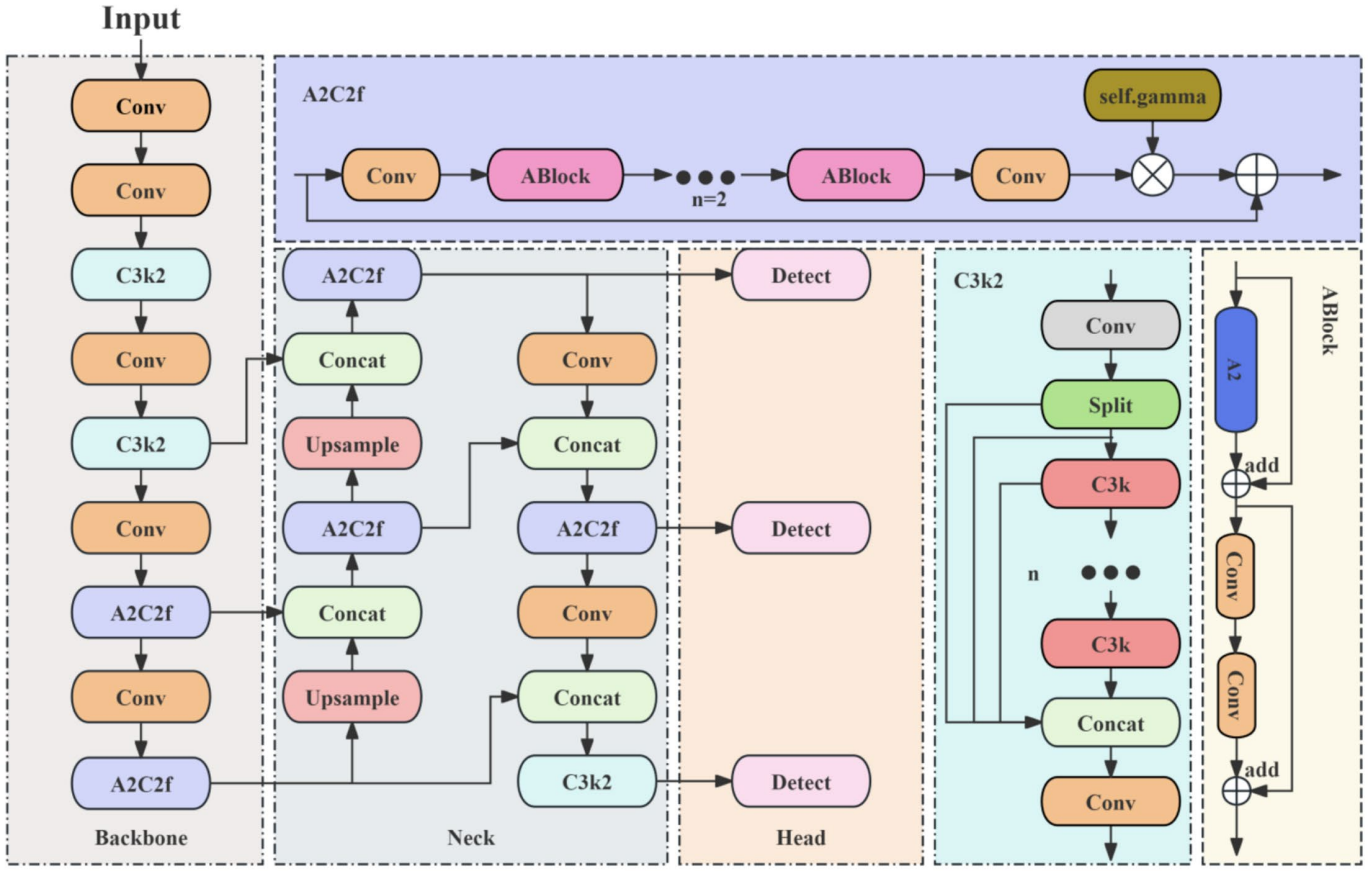
Schematic of the YOLO12 model architecture.

Given the practical demand for lightweight design and efficient inference in clinical deployment, the most lightweight variant, YOLO12n, is selected as the baseline in this study. Its structure is illustrated in Figure 1.

## 3 FAD-YOLO

**Figure 2:**
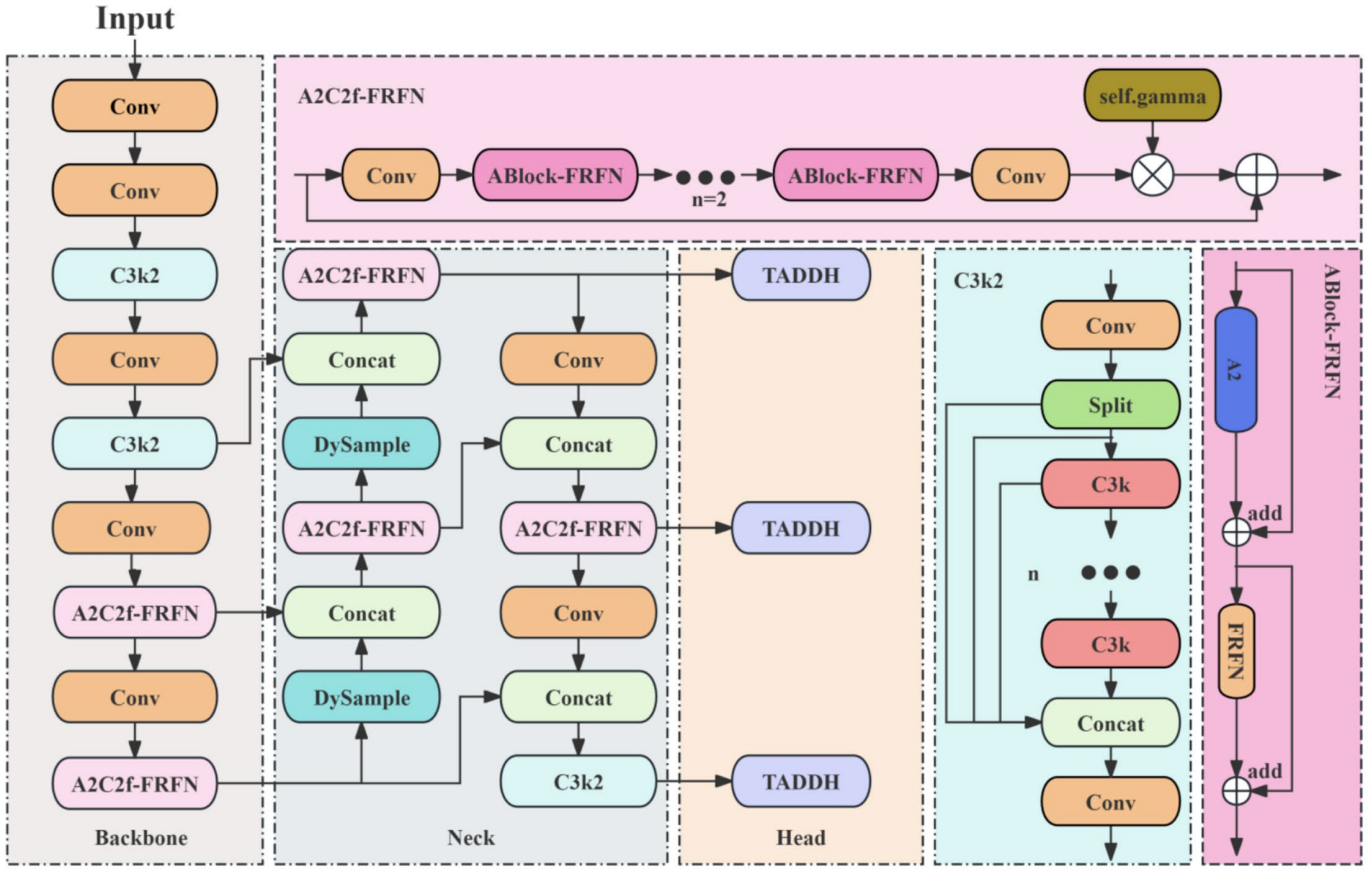
Schematic of the FAD-YOLO model architecture.

The proposed FAD-YOLO is built upon YOLO12n and incorporates targeted modifications at three critical locations, namely the A2C2f module of the backbone, the upsampling pathway of the neck, and the detection head, so as to address the three domain-adaptation limitations of YOLO12n in the AIS/MIA discrimination task on pulmonary CT. The overall architecture is shown in Figure 2, and the design rationale and implementation details of each improved module are described in the following subsections.

### 3.1 A2C2f-FRFN feature refinement module

**Figure 3:**
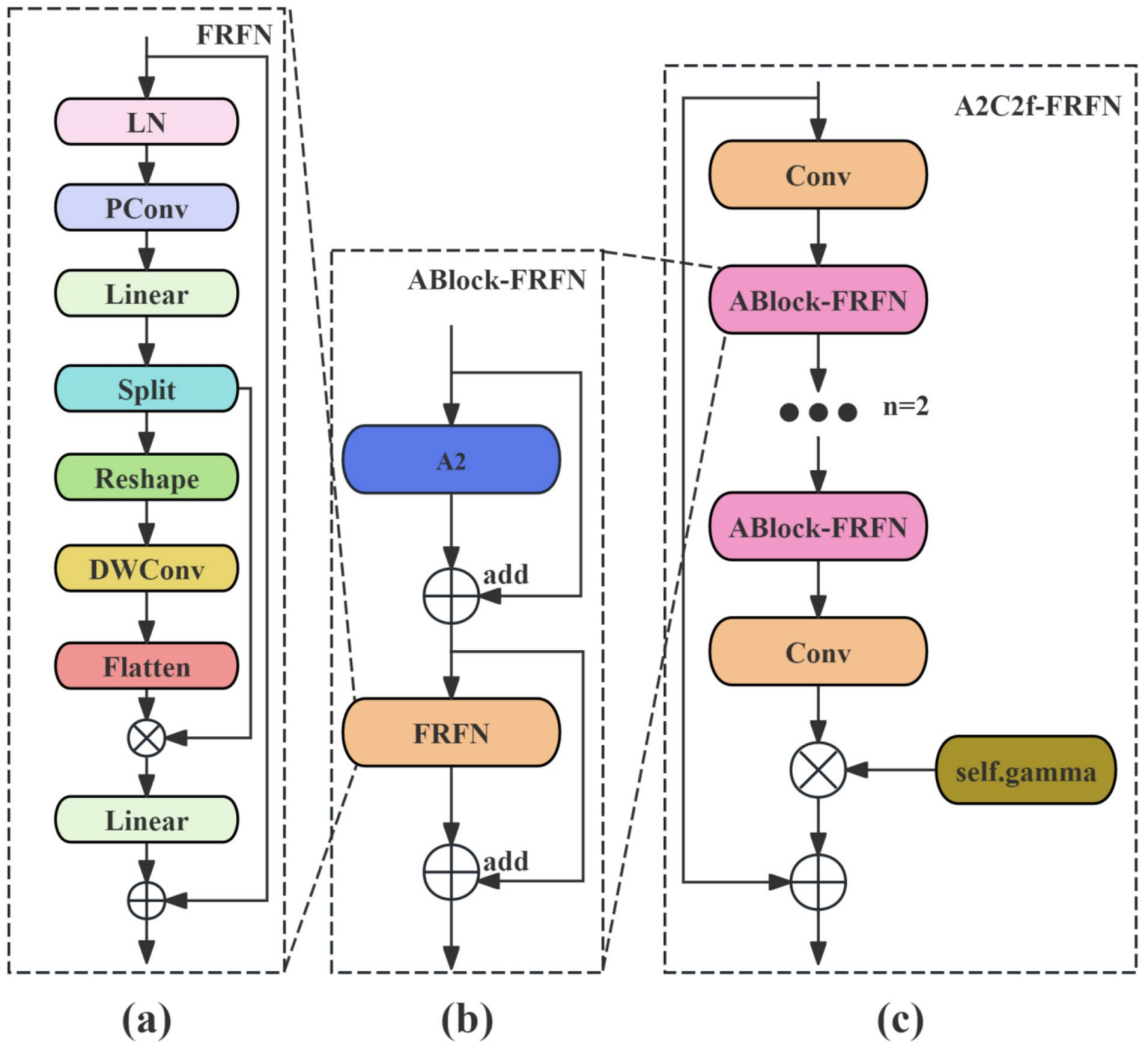
Architecture of A2C2f-FRFN: **(a)** FRFN; **(b)** ABlock-FRFN; **(c)** A2C2f-FRFN.

The core feature aggregation unit of the YOLO12n backbone, A2C2f, consists of Area Attention layers and standard feed-forward networks (FFNs) stacked in an alternating fashion, with an outer CSP-style branch that facilitates gradient flow. Although Area Attention performs local attention modeling at the regional scale, the standard FFN within A2C2f is composed of two 1 × 1 convolutional layers and a GELU activation, performing only linear transformations along the channel dimension and lacking explicit characterization of local spatial structure. For AIS/MIA discrimination on pulmonary CT, GGNs are small in size, exhibit diffuse boundaries, and present subtle density differences, while critical discriminative cues such as spiculation, lobulation, and minute internal solid components correspond to fine-grained local patterns that occupy only a small fraction of the feature response. The standard FFN treats all channels uniformly and provides no mechanism to selectively emphasize discriminative activations or suppress redundant background responses, making it difficult to highlight the subtle local details essential for distinguishing AIS from MIA. Accordingly, this study replaces the standard FFN in A2C2f with the Feature Refinement Feed-forward Network (FRFN), forming the A2C2f-FRFN feature refinement module, as illustrated in Figure 3.

The core idea of FRFN, originally proposed within the Adaptive Sparse Transformer (AST) framework, is to selectively amplify salient activations while dampening redundant responses through a lightweight, content-adaptive interaction. As illustrated in Figure 3a, let the input feature map be X. The input first passes through layer normalization (LN) and a partial convolution (PConv), yielding F₁ = PConv(LN(X)). The result is then projected by a linear layer and split along the channel dimension into two complementary components: [Fa, Fᵇ] = split(Linear(F₁)), where Fa carries activation weights and Fᵇ serves as a modulating signal. To capture local spatial cues, Fᵇ is reshaped, processed by a depthwise convolution (DWConv), and flattened to obtain Fᵇ′ = Flatten(DWConv(Reshape(Fᵇ))). An adaptive interaction is performed via element-wise multiplication, R(F) = Fa ⊗ Fᵇ′, which emphasizes structure-consistent features while suppressing responses irrelevant to object boundaries or semantics. Finally, a linear projection with a residual connection produces Y = Linear(R(F)) + X. Through this gated, content-adaptive pathway, FRFN strengthens fine-grained spatial representation without any explicit frequency-domain transform; the enhancement of detail-related responses is achieved through multiplicative gating rather than subtractive frequency decomposition.

Building on this pathway, the ABlock-FRFN unit shown in Figure 3b integrates an Area Attention layer and an FRFN sub-network, both wrapped by residual connections. Area Attention partitions the feature map into local regions and computes attention within each region, providing approximately global modeling at linear complexity, while FRFN applies the gated refinement above to the attention output, suppressing residual redundancy and reinforcing discriminative spatial details.

The complete A2C2f-FRFN module is illustrated in Figure 3c. It retains the CSP topology of the original A2C2f: the input is projected by a 1 × 1 convolution and split into a main branch and a shortcut branch; the main branch passes through n stacked ABlock-FRFN units, whose intermediate outputs are preserved. All responses are concatenated along the channel dimension, fused by a 1 × 1 convolution, scaled by a lightweight modulation operator, and added to the input to form the final output. Compared with the standard A2C2f, the FFN inside every Transformer subunit is replaced by FRFN, while all other residual and normalization layers remain unchanged. Consequently, Area Attention contributes region-scale global–local modeling, FRFN performs gated channel-wise refinement, and the surrounding CSP structure preserves multi-path gradient flow, jointly enhancing discriminative capacity for the low-contrast density differences and fine-grained edge cues critical to AIS/MIA discrimination. Owing to the low overhead of partial and depthwise convolutions, the increase in parameters and FLOPs relative to the original A2C2f is only marginal.

### 3.2 DySample dynamic upsampling module

Ground-glass nodules on CT images exhibit continuous density gradients and naturally graded transitions toward the surrounding pulmonary parenchyma; any upsampling operator that disrupts this continuity will impair fine-grained AIS/MIA discrimination. The neck FPN-PAN pathway of YOLO12n adopts standard nearest-neighbor interpolation as the upsampling operator, which simply replicates neighboring pixels at integer ratios, contains no learnable parameters, and cannot perceive the local semantics of the input feature. When applied to GGNs, which are lesions highly sensitive to density transitions and minute solid components, such pixel-replication-based enlargement discretizes the otherwise continuous density gradient and further blurs the already diffuse nodule contour, with particularly adverse effects on small AIS lesions.

**Figure 4:**
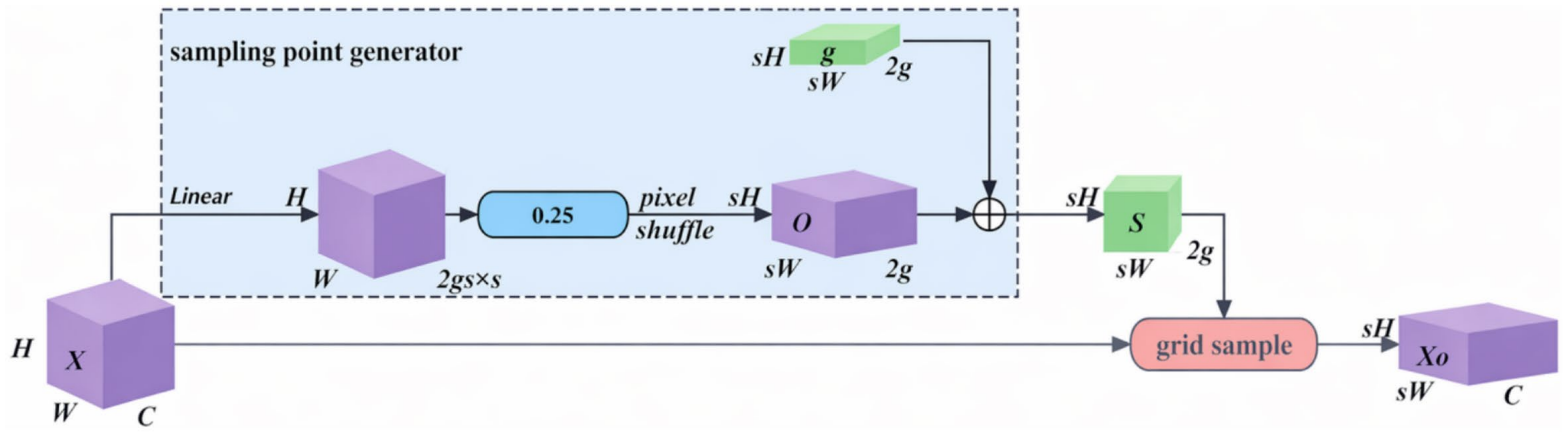
Architecture of the DySample upsampling module.

Accordingly, this study employs DySample, proposed by Liu et al. (27), to replace nearest-neighbor upsampling in the FPN-PAN, with its overall structure shown in Figure 4. Unlike conventional approaches that directly predict high-resolution pixel values, DySample reformulates upsampling as “locating appropriate sampling positions on the original feature map”: a two-dimensional offset is learned for each output position, and the input feature map is then resampled in a content-adaptive, continuous-domain manner, so that the enlargement process is no longer constrained to fixed neighborhood templates.

Given an input feature X with C channels and spatial size H × W and an upsampling ratio s, the computation in DySample is divided into two stages: offset prediction and resampling. The offset-prediction stage is performed by a “sampling-point generator”: X first undergoes a linear transformation to yield an offset field with 2gs^2^ channels (where g denotes the number of groups), which is then multiplied by a static range factor of 0.25 to constrain the offset magnitude. After pixel shuffle, the channel information is redistributed into the high-resolution spatial dimensions sH × sW, producing a position-wise two-dimensional offset tensor O. Adding O to the regular grid g yields a content-dependent set of sampling coordinates S. In the resampling stage, S is used as an index, and a differentiable bilinear sampling operator (grid_sample in PyTorch) reads the corresponding sub-pixel values from X to output a high-resolution feature Xo of size sH × sW × C. The entire pipeline is end-to-end differentiable, and the offset predictor is jointly trained with the main network.

**Figure 5:**
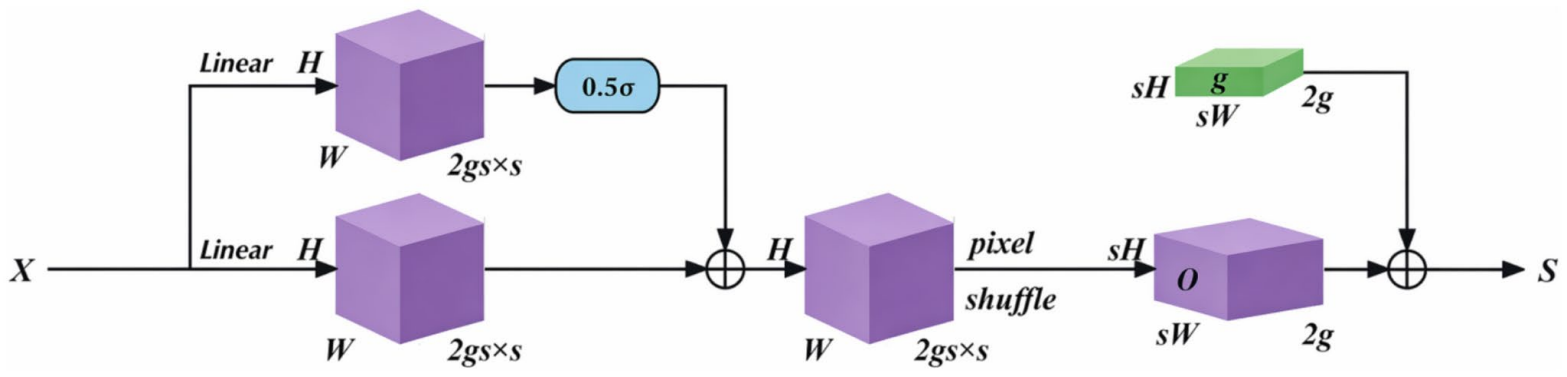
Dynamic range factor.

As an optional enhancement, DySample also supports the replacement of the static range factor in Figure 4 with a point-wise dynamic range factor, whose structure is illustrated in Figure 5. This mechanism appends a parallel linear projection alongside the original offset branch to generate a per-point scale from the input feature. Following sigmoid activation, the result is multiplied by 0.5 to maintain an amplitude upper bound equivalent to the static formulation, and is then multiplied point-wise with the original offset field; pixel shuffle is subsequently applied to obtain the offset tensor O. In this way, each spatial position can adaptively modulate its offset magnitude according to its local semantics, at the cost of additional overhead from the supplementary linear branch.

Balancing accuracy and computational cost, the present work adopts the “LP-style + static range factor + grouped offsets” DySample configuration in the neck pathway of FAD-YOLO. The LP (Linear-PixelShuffle) variant places offset prediction prior to pixel shuffle, completing the pipeline with lower peak memory usage; the static range factor obviates the additional computation of the dynamic branch in Figure 5; and the grouped offsets divide channels into several groups that share a common offset, compressing the parameter count of the offset predictor to 1/g of its per-channel counterpart. Because the offset predictor consists only of a single convolution and a single pixel-shuffle operation, the additional parameters and computation introduced into the neck remain minimal. After replacing nearest-neighbor interpolation with DySample, the neck upsampling shifts from content-agnostic pixel replication to a geometric transformation that is responsive to input content: along the outer edges of the nodule, the learned offsets converge in the direction of the density gradient, preserving the boundary transition zone that is otherwise smeared out by static replication; within the nodule, the offset field becomes smooth, retaining the imaging characteristic of “density gradually decreasing from the center to the periphery,” which is decisive for AIS/MIA discrimination, and thereby providing the detection head with multi-scale inputs that contain more complete boundary and density information.

### 3.3 TADDH task-aligned dynamic detection head

**Figure 6:**
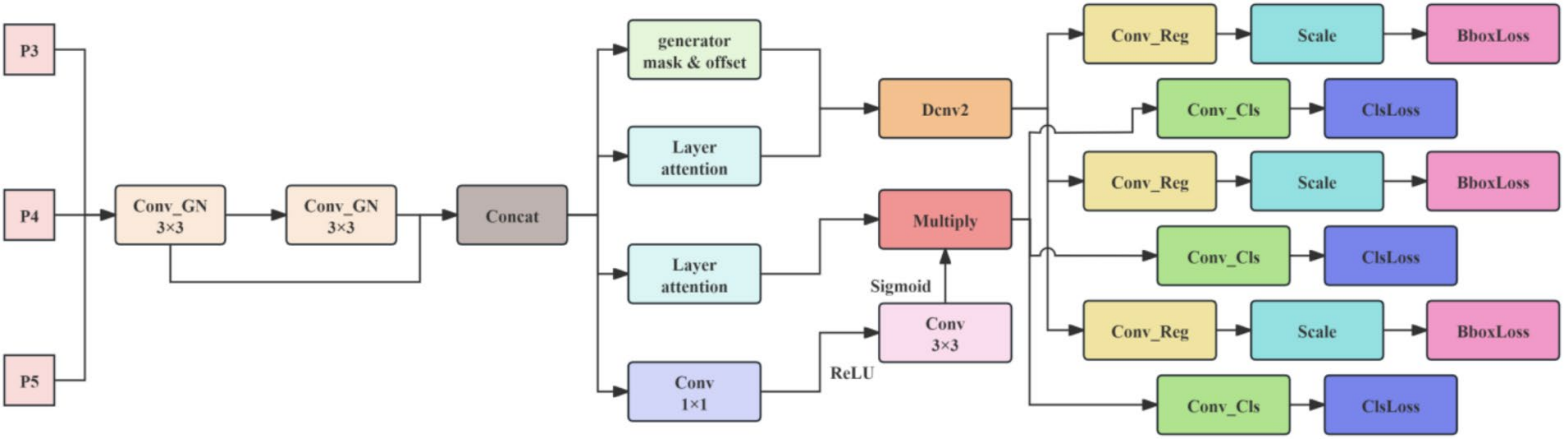
Architecture of the TADDH task-aligned dynamic detection head.

The detection head of YOLO12n inherits the decoupled-head structure used since YOLOX. The classification and regression branches each process the input features through an independent sequence of a 3 × 3 convolution followed by a 1 × 1 convolution, and, respectively, output class probabilities and bounding-box offsets. Although this structure decouples the two task outputs, their spatial feature extraction is performed independently, so the location emphasized by the classification branch may not coincide with that relied upon by the regression branch, giving rise to a classification and localization spatial misalignment problem. For AIS/MIA discrimination, in which GGNs exhibit varied morphology and blurred boundaries, this misalignment amplifies two typical errors, namely redundant boxes with high classification confidence but biased regression boundaries, and missed detections that are accurately localized yet suppressed by insufficient classification response. Accordingly, this study adopts the Task-Aligned Dynamic Detection Head (TADDH), which builds on the task-alignment principle of TOOD (28) and the attention-based dynamic-head design of Dynamic Head (29), together with the deformable sampling of DCNv2 (already cited), to replace the decoupled head of YOLO12n. Its structure is illustrated in Figure 6.

TADDH is constructed around three coordinated components, namely a shared group-normalized convolution that aligns the source features of both tasks, a task-decomposition pathway in which deformable sampling is applied within the localization branch, and a layer-attention mechanism that performs task-specific feature reweighting. Specifically, multi-scale features from the neck first pass through a shared 3 × 3 convolution with group normalization (Conv_GN), which extracts a common set of joint features and reduces parameters relative to a standard decoupled head. From these joint features, two complementary pathways are derived. In the localization pathway, a feature corrector generates the offsets and modulation masks required by deformable convolution v2 (DCNv2) (30), which then adaptively shifts its sampling positions away from a fixed rectangular grid so that the kernel conforms to the actual GGN morphology. In parallel, a layer-attention mechanism compresses the joint features through global average pooling (GAP) and produces channel-wise weights via a sigmoid-activated transformation. Its mathematical expression is given in Equation 1:


$$w=\sigma ({\mathrm{FC}}_{2}(\delta ({\mathrm{FC}}_{1}(x^{\text{inter}}))))$$ (1)

Where FC₁(⋅) and FC₂(⋅) denote two fully connected layers; σ denotes the sigmoid activation and δ the ReLU activation; and $x^{\text{inter}}$ denotes the concatenated feature obtained by applying average pooling to $X^{\text{inter}}$ .

These weights are used to recalibrate the deformable-sampled features before they enter the regression output (Conv_Reg) and the bounding-box scaling layer. In the classification pathway, the joint features are reweighted by a parallel layer-attention branch and combined with a refined response from a 1 × 1 convolution, ReLU activation, 3 × 3 convolution, and sigmoid gating, then projected through a classification convolution (Conv_Cls) to produce class probabilities. The deformable convolution is therefore deployed only within the localization branch rather than placed before both, while the two pathways share the upstream Conv_GN and exchange information through complementary layer-attention reweighting, reducing detection-head parameters and GFLOPs while resolving spatial focus differences at the source.

With respect to AIS/MIA discrimination, the advantages of TADDH manifest in three aspects. First, the shared Conv_GN stage anchors classification and regression to consistent joint features, alleviating the inconsistency of high classification confidence coupled with low localization quality on blurred boundaries. Second, the deformable convolution within the localization branch conforms its sampling grid to the actual GGN morphology, producing tighter bounding boxes for lesions with diffuse or irregular contours. Third, the layer-attention mechanisms reweight joint features in a task-specific manner, biasing the classification branch toward channels sensitive to histological differences between AIS and MIA and the localization branch toward channels supporting accurate boundary regression, while parameter sharing through Conv_GN keeps the head leaner and amenable to deployment on clinical edge devices.

## 4 Experiments and result analysis

### 4.1 Dataset and preprocessing

The medical imaging data were drawn from two independent datasets. Dataset 1 comprised pulmonary CT images retrospectively collected at the People’s Hospital Affiliated with Fujian University of Traditional Chinese Medicine. Data collection was approved by the institutional ethics committee (approval no. YJS2025-147-02), and the requirement for informed consent was waived. This dataset was used for model training, validation, and internal testing. Eligible cases were lesions that had been surgically resected and pathologically confirmed as AIS or MIA between December 2019 and June 2025. The inclusion criteria were: (1) a chest CT scan completed within 1 month before surgery; (2) postoperative pathological confirmation of AIS or MIA; (3) a maximum nodule diameter < 3.0 cm; and (4) thin-section reconstruction with a slice thickness of 1 mm. The exclusion criteria were: (1) coexisting pulmonary malignancies or mixed pathological types; and (2) preoperative radiotherapy or chemotherapy. Separately, images with severe artifacts were removed during slice-level quality control rather than being treated as a subject-level exclusion criterion; the resulting number of retained images is reported below. In total, 225 pathologically confirmed AIS and MIA lesions from 198 patients were included.

**Figure 7:**
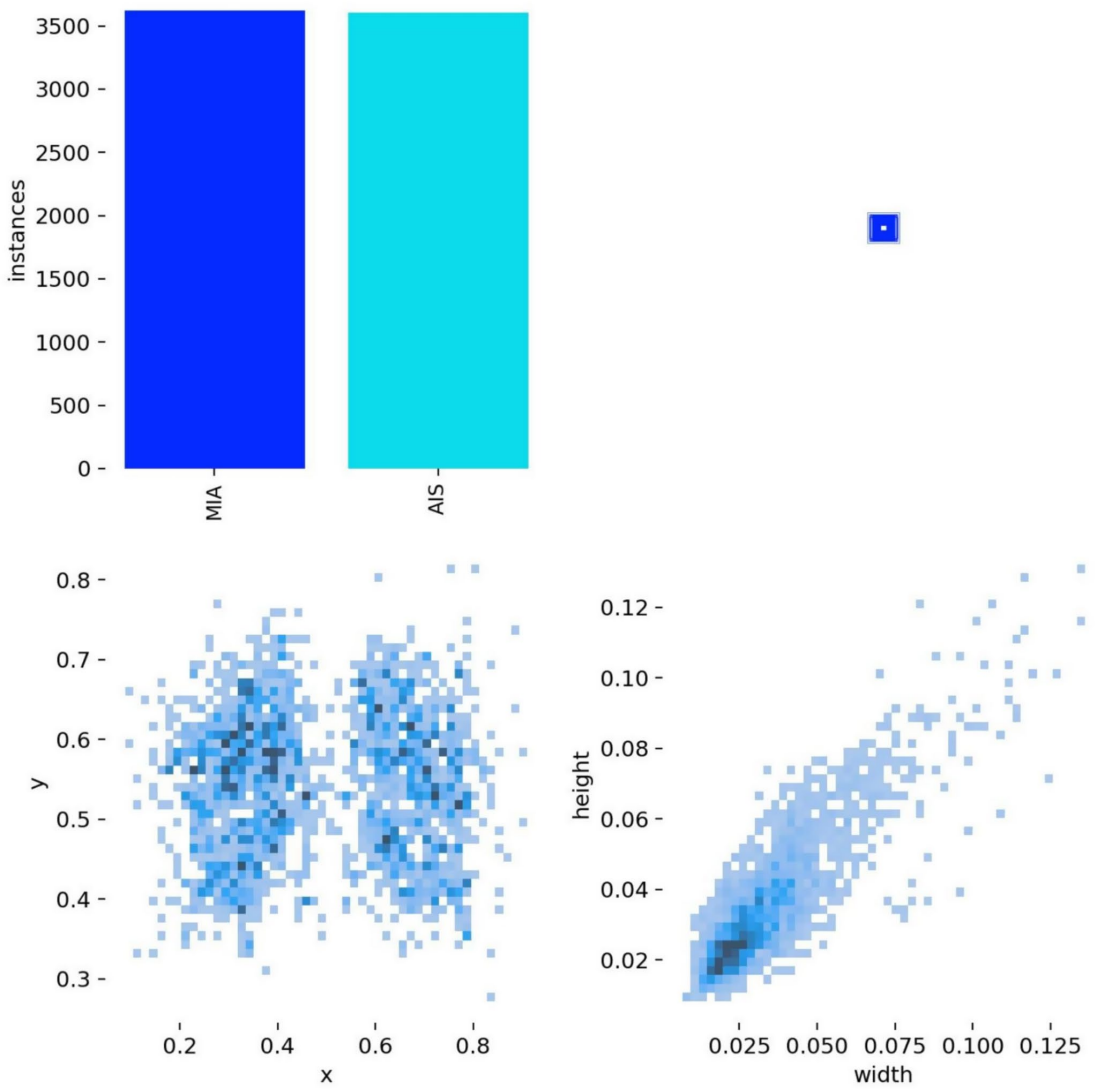
Annotation status of the training set.

**Figure 8:**
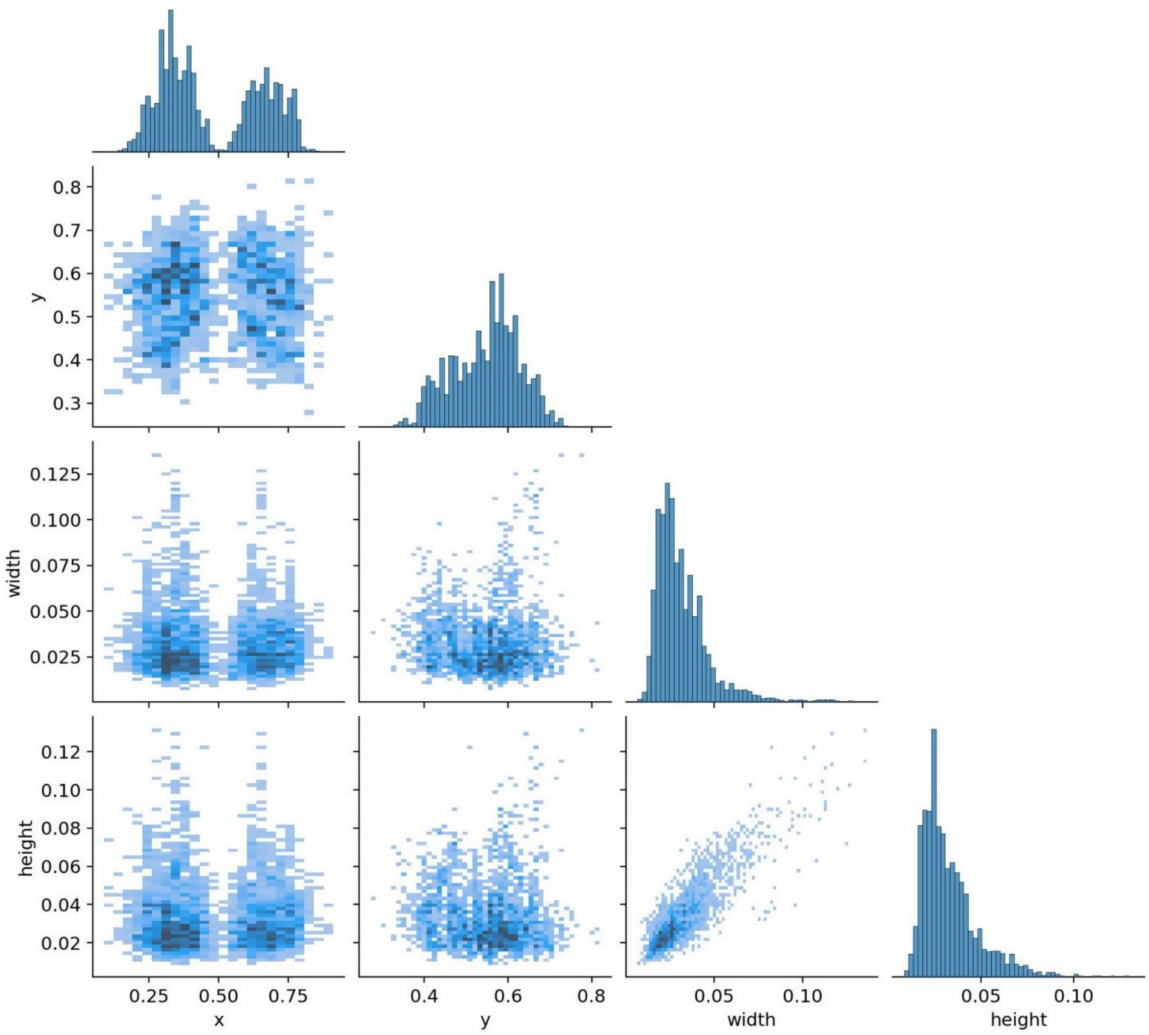
Annotation correlation map of the training set.

DICOM images were exported from the PACS system, windowed using a lung window setting (W = 1,500 HU, L = −600 HU), linearly mapped to an 8-bit grayscale range (0–255), and uniformly converted to PNG images at a resolution of 512 × 512. After slice-level screening and quality control, 2,122 images from the 198 patients were retained (1,058 AIS and 1,064 MIA), forming a class-balanced dataset; as detailed below, all subsequent partitioning of these images was performed at the patient level. Lesions were annotated as bounding boxes by two radiologists using LabelImg, and the annotations were subsequently cross-checked and reconciled by a senior radiologist (associate chief physician or higher) into a single consensus standard. The annotation distribution and correlation of the training set are shown in Figures 7, 8.

A five-fold cross-validation strategy was adopted for data partitioning. From Dataset 1, a cross-validation pool of 1,805 images (168 patients, 191 lesions) was randomly selected and evenly partitioned into five folds of 361 images each. In each of the five iterations, four folds were combined for training (1,444 images) and the remaining fold was used for validation (361 images); the per-fold composition is detailed in Table 1. The remaining 317 images (30 patients, 34 lesions) were held out as an independent internal test set and kept identical across all folds. To prevent data leakage, all images were split at the patient level, so that no patient contributed images to more than one subset. All images were uniformly resized to 640 × 640 and normalized prior to training and inference. Ablation experiments were evaluated on the validation folds to support statistical analysis; specifically, paired t-tests were performed on the per-fold mAP@50 values. Comparative experiments, by contrast, were evaluated on the single held-out 317-image independent test set to ensure an unbiased, leakage-free benchmark; because each model yields a single value on this fixed test set, the per-fold paired t-test used in the ablation is not applicable to the cross-network comparison.

**Table 1:** Per-fold data partition of Dataset 1 under five-fold cross-validation.

Fold	Train patients	Train lesions	Train images	Val patients	Val lesions	Val images
1	134	152	1,444	34	39	361
2	134	153	1,444	34	38	361
3	134	153	1,444	34	38	361
4	135	153	1,444	33	38	361
5	135	153	1,444	33	38	361

Because the data were split at the patient level, the per-fold patient and lesion counts vary slightly across folds, whereas the image counts are fixed at 1,444 for training and 361 for validation in every fold. The five validation folds are mutually exclusive and together sum to 168 patients and 191 lesions, corresponding exactly to the cross-validation pool. The independent internal test set of 317 images (30 patients, 34 lesions) was held out from this pool and used identically across all five folds. After offline augmentation, which was applied to the training set only, each fold’s training set was expanded from 1,444 to 7,220 images.

Dataset 2 was obtained from the publicly available Mendeley Data repository (Mendeley Data, https://data.mendeley.com/datasets/h7b4ryrbzw) (31) and served as an external independent test set for cross-dataset generalization assessment. It differs substantially from Dataset 1 in imaging acquisition workflows, patient composition, and scanner parameters. CT images meeting the AIS/MIA discrimination criteria were screened and annotated using the same procedure as for Dataset 1, ultimately yielding an external test set of 300 annotated images (150 AIS and 150 MIA). Dataset 2 was used solely for generalization assessment and did not participate in any training or validation process.

**Figure 9:**
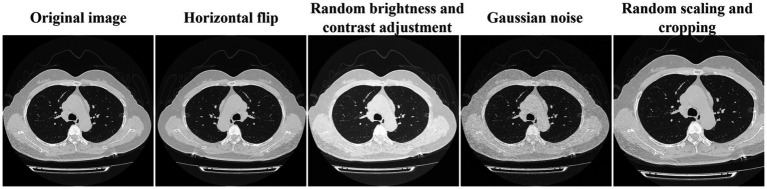
Comparison of the four data augmentation operations.

Offline data augmentation was applied only to the training set of Dataset 1; no augmentation was performed on the validation set, the internal test set, or Dataset 2. To balance spatial diversity with grayscale fidelity, four augmentation operations were employed: (1) horizontal flipping to simulate the distribution of lesions across the left and right lung fields; (2) random brightness/contrast adjustment (±15%) to simulate variability in scanning parameters; (3) additive Gaussian noise to simulate quantum noise under different dose conditions; and (4) random scaling and cropping (0.8–1.2×) to reflect variations in field of view and lesion scale. Representative results are compared in Figure 9. Four augmented copies were generated from each original image, expanding the training set to 7,220 images, fivefold its original size.

### 4.2 Experimental environment and hyperparameter settings

All experiments were conducted on the same hardware platform. The operating system was Windows 11, the processor was an Intel(R) Core(TM) Ultra 9275HX, the GPU was an NVIDIA GeForce RTX 5070 Laptop GPU, and the system memory was 32 GB. The deep learning framework was PyTorch 2.8.0 with CUDA 12.9, and Python 3.9.23. The principal training hyperparameters are listed in Table 2 and were kept identical across all experiments to ensure fair comparison. The hyperparameters follow widely adopted defaults for the YOLO and RT-DETR families rather than task-specific tuning: the AdamW optimizer was used with its default momentum coefficient of 0.9; the image size (640 × 640), batch size (16), and 100-epoch schedule were chosen to balance convergence against the memory of the deployment-class GPU; and the same settings were applied to every model so that performance differences reflect architecture rather than optimisation. No per-model hyperparameter search was performed.

**Table 2:** Model hyperparameter settings.

Image size	Batch size	Epochs	Optimizer	momentum	NMS IoU
640 × 640	16	100	AdamW	0.9	0.7

### 4.3 Evaluation metrics

Detection performance was evaluated along two dimensions: accuracy and model complexity. The accuracy metrics included precision (P), recall (R), and mean average precision (mAP), defined in Equations (2–5). Model complexity was characterized by the parameter count (Params, in M) and the floating-point operations (GFLOPs), which, respectively, reflect storage and computational cost; lower values of both metrics indicate a more lightweight model that is better suited to deployment on resource-constrained clinical devices.


$$P=\frac{\mathrm{TP}}{\mathrm{TP}+\mathrm{FP}}$$ (2)

$$\begin{array}{c} R=\frac{\mathrm{TP}}{\mathrm{TP}+\mathrm{FN}} \end{array}$$ (3)

$$\begin{array}{c} \mathrm{AP}=\int_{0}^{1}P(R)\mathrm{dR} \end{array}$$ (4)

$$\begin{array}{c} \mathrm{mAP}=\frac{1}{N}\sum_{i=1}^{N}{\mathrm{AP}}_{i} \end{array}$$ (5)

Here, TP (true positive), FP (false positive), and FN (false negative) follow standard definitions. AP is obtained by integrating the precision–recall curve for a single class and reflects the detection capability for that class; mAP is the arithmetic mean of AP across all classes and quantifies overall detection quality. Two mAP variants under different IoU thresholds are reported in this paper: mAP@50, the mean average precision at IoU = 0.50, which emphasizes localization robustness; and mAP@50–95, the mean average precision averaged over IoU values from 0.50 to 0.95 in steps of 0.05, which provides a more stringent evaluation of performance under varying localization tolerances.

### 4.4 Ablation study

**Figure 10:**
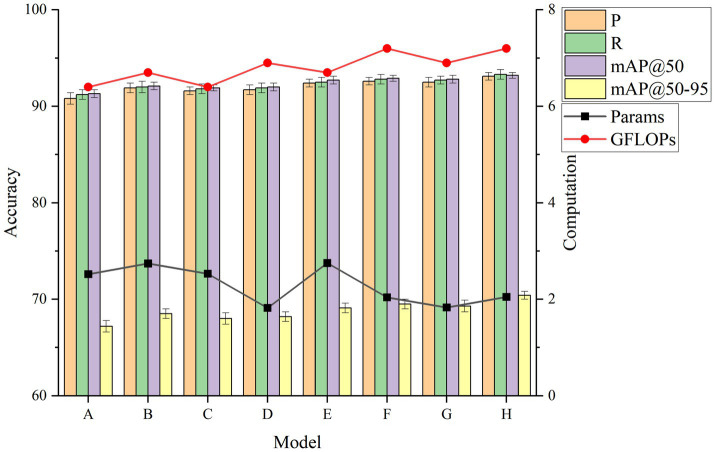
Comparative results of the ablation study.

To validate the independent contribution of each module, an ablation study was conducted on the validation folds of Dataset 1, with YOLO12n serving as the baseline. The hardware, hyperparameters, and five-fold cross-validation protocol were kept identical. The metrics included P, R, mAP@50, mAP@50–95, Params, and GFLOPs. The three improvements were introduced in sequence: (1) replacing the standard FFN within the A2C2f module with FRFN; (2) substituting nearest-neighbor interpolation in the FPN-PAN upsampling pathway with DySample; and (3) replacing the decoupled head of YOLO12 with the TADDH task-aligned dynamic detection head. The results are summarized in Table 3 and Figure 10.

**Table 3:** Ablation study results.

Model	FRFN	DySample	TADDH	P/%	R/%	mAP@50/%	mAP@50–95/%	Params/10^6^	GFLOPs
A	–	–	–	90.8 ± 0.6	91.2 ± 0.5	91.3 ± 0.4	67.2 ± 0.6	2.52	6.4
B	✓	–	–	91.9 ± 0.5	92.0 ± 0.6	92.1 ± 0.4	68.5 ± 0.5	2.74	6.7
C	–	✓	–	91.6 ± 0.4	91.8 ± 0.5	91.9 ± 0.3	68.0 ± 0.6	2.53	6.4
D	–	–	✓	91.7 ± 0.5	91.9 ± 0.5	92.0 ± 0.4	68.2 ± 0.5	1.82	6.9
E	✓	✓	–	92.4 ± 0.4	92.5 ± 0.5	92.7 ± 0.4	69.1 ± 0.5	2.75	6.7
F	✓	–	✓	92.6 ± 0.4	92.8 ± 0.5	92.9 ± 0.3	69.5 ± 0.5	2.04	7.2
G	–	✓	✓	92.5 ± 0.5	92.7 ± 0.4	92.8 ± 0.4	69.3 ± 0.6	1.83	6.9
H	✓	✓	✓	93.1 ± 0.4	93.3 ± 0.5	93.2 ± 0.3	70.4 ± 0.4	2.05	7.2

Values are reported as mean ± standard deviation across the five cross-validation folds.

Each module produced a performance gain when introduced individually, and their combination exhibited a markedly synergistic effect. On the validation set, the YOLO12n baseline attained *p* = 90.8 ± 0.6%, R = 91.2 ± 0.5%, mAP@50 = 91.3 ± 0.4%, and mAP@50–95 = 67.2 ± 0.6%, with 2.52 × 10^6^ parameters and 6.4 GFLOPs, serving as the reference benchmark.

With FRFN introduced alone, mAP@50 increased from 91.3 to 92.1%, and mAP@50–95 rose from 67.2 to 68.5%, confirming the value of coupling gated multiplicative refinement with depthwise spatial filtering for characterizing subtle density differences and fine-grained edge cues in low-contrast GGNs; the lightweight design, based on depthwise separable convolution and channel splitting, resulted in only marginal increases, with parameters rising from 2.52 × 10^6^ to 2.74 × 10^6^, and GFLOPs from 6.4 to 6.7. With DySample introduced alone, mAP@50 rose to 91.9% and mAP@50–95 to 68.0%, with virtually no change in parameters or GFLOPs; this indicates that replacing nearest-neighbor interpolation with content-aware learnable offsets preserves GGN boundary sharpness and the integrity of internal density transitions at almost no additional cost. With TADDH introduced alone, parameters were reduced from 2.52 × 10^6^ to 1.82 × 10^6^, while GFLOPs increased only modestly from 6.4 to 6.9, with the slight rise attributable to the small additional computation introduced by the deformable convolution within the localization branch; mAP@50 still rose to 92.0% and mAP@50–95 to 68.2%, confirming the effectiveness of the shared Conv_GN, deformable sampling, and layer-attention reweighting in alleviating classification–localization misalignment, and demonstrating that the joint-feature sharing introduced through the upstream Conv_GN substantially reduces the storage cost of the detection head.

Among the pairwise combinations, FRFN + DySample simultaneously enhanced the discriminative-feature selectivity of the backbone and the content awareness of the neck upsampling, achieving mAP@50 = 92.7% and mAP@50–95 = 69.1%, both representing further gains over the single-module variants. The FRFN + TADDH combination exploited the complementary advantages of detail enhancement in the backbone and task alignment in the detection head, yielding accuracy improvements together with substantial parameter compression: Params dropped to 2.04 × 10^6^, a 19.0% decrease relative to the baseline, while GFLOPs rose to 7.2. The DySample + TADDH combination, which strengthened upsampling quality and task alignment at the neck and head, respectively, achieved mAP@50 = 92.8% and mAP@50–95 = 69.3%. All pairwise combinations outperformed the single-module variants, demonstrating that the three modules assume differentiated functions at distinct positions within the network and exhibit favorable mutual compatibility.

With all three improvements integrated, FAD-YOLO achieved the best overall performance: *p* = 93.1 ± 0.4%, R = 93.3 ± 0.5%, mAP@50 = 93.2 ± 0.3%, and mAP@50–95 = 70.4 ± 0.4%, corresponding to gains of 2.3, 2.1, 1.9, and 3.2 percentage points over the baseline, respectively. The notable improvement on mAP@50–95 indicates a markedly larger gain under stringent localization criteria, which is consistent with the combined design objective of the three modules in boundary perception and task alignment. Concurrently, parameters decreased from 2.52 × 10^6^ to 2.05 × 10^6^, while GFLOPs rose only modestly from 6.4 to 7.2, demonstrating that FAD-YOLO achieves substantial parameter compression alongside accuracy gains and is therefore deployable on resource-constrained devices. Paired t-tests on per-fold mAP@50 values showed that FAD-YOLO significantly outperformed the baseline (*p* = 0.0041) and was also statistically superior to each single-module variant (FRFN: *p* = 0.0104; DySample: *p* = 0.0128; TADDH: *p* = 0.0089; all *p* < 0.05).

### 4.5 Comparative experiments

To further verify the overall advantages of FAD-YOLO, it was compared with YOLOv5n, YOLOv8n, YOLO11n, RT-DETR-R18, RT-DETR-R50, and the YOLO12n baseline on the independent test set of Dataset 1. These baselines comprise several lightweight one-stage detectors (YOLOv5n, YOLOv8n, YOLO11n, and YOLO12n) and the Transformer-based RT-DETR family at two backbone capacities (R18 and R50), enabling a fair assessment of FAD-YOLO across detection paradigms and across a wide range of model sizes. The classical two-stage detector Faster R-CNN was not included as a baseline, since comparing it at its default settings would conflate the two-stage detection paradigm and its training configuration with architectural merit and would therefore be uninformative for the lightweight, end-to-end design targeted in this study. All models, including RT-DETR-R50, were trained and evaluated under identical data splits, training strategies, and hyperparameters, with the same random seed where applicable. Inference speed was measured on the same NVIDIA GeForce RTX 5070 Laptop GPU at an input size of 640 × 640, with 50 warm-up iterations and 300 repetitions to ensure stability. The results are reported in Table 4.

**Table 4:** Comparative experimental results across networks.

Model	P/%	R/%	mAP@50/%	mAP@50–95/%	Params/10^6^	GFLOPs	FPS
YOLOv5n	89.6	89.3	89.5	63.8	2.51	7.2	154
YOLOv8n	90.5	90.2	90.3	64.4	3.01	8.2	147
YOLO11n	91.0	90.8	90.9	65.6	2.58	6.4	142
YOLO12n	91.4	91.6	91.8	67.5	2.52	6.4	138
RT-DETR-R18	91.8	92.0	91.6	69.5	20.08	58.3	83
RT-DETR-R50	93.1	92.6	92.7	70.4	42.77	130.5	52
FAD-YOLO	93.8	93.4	93.6	70.8	2.05	7.2	131

As shown in Table 4, FAD-YOLO leads all evaluated detectors on the two core accuracy metrics, with an mAP@50 of 93.6% and an mAP@50–95 of 70.8%.

Compared with the YOLO nano series (YOLOv5n, YOLOv8n, YOLO11n, and YOLO12n), FAD-YOLO leads by 1.8–4.1 percentage points on mAP@50 and by 3.3–7.0 percentage points on mAP@50–95. Although the YOLO nano series exhibits very small parameter counts (2.51–3.01 M), GFLOPs in the 6.4–8.2 range, and high throughput (138–154 FPS), its general-purpose nano architecture is optimized for natural images and lacks targeted modeling for low-contrast GGN details, which limits its accuracy on fine-grained tasks such as AIS/MIA discrimination and hampers reliable extraction of discriminative features in low-contrast boundaries and density-heterogeneous regions. Compared with RT-DETR-R18, FAD-YOLO achieves a slightly higher mAP@50–95 (70.8 vs. 69.5) with approximately one-tenth the parameter count (2.05 vs. 20.08 M) and about one-eighth the GFLOPs (7.2 vs. 58.3), and substantially higher inference speed (131 vs. 83 FPS), demonstrating the superior structural efficiency of the YOLO12n architecture under equivalent accuracy. Against the substantially larger RT-DETR-R50 variant (42.77 M parameters, 130.5 GFLOPs, 52 FPS), FAD-YOLO attains slightly higher accuracy on every metric (mAP@50 93.6% vs. 92.7%; mAP@50–95 70.8% vs. 70.4%) while using approximately one-twentieth of the parameters, about one-eighteenth of the GFLOPs, and roughly 2.5 times the inference speed, further underscoring the favourable accuracy–efficiency trade-off of the proposed design.

Relative to the YOLO12n baseline, FAD-YOLO achieves an 18.7% reduction in parameters (with GFLOPs rising slightly from 6.4 to 7.2) while improving mAP@50 and mAP@50–95 by 1.8 and 3.3 percentage points, respectively; inference speed decreases marginally from 138 FPS to 131 FPS. This decrease is mainly attributable to the irregular memory-access patterns of DCNv2 deformable convolution within TADDH, compounded by the small operator-launch overhead introduced by DySample dynamic sampling and the additional FRFN branch, which together yield a wall-clock latency higher than that suggested by FLOPs alone; nevertheless, FAD-YOLO remains well above the 30-FPS real-time threshold and fully satisfies the throughput requirements of clinical deployment. In summary, with 2.05 M parameters, 7.2 GFLOPs, and 131 FPS, FAD-YOLO strikes a favorable balance among accuracy, lightweight design, and real-time performance, suggesting that it may serve as a candidate for further prospective validation in AIS/MIA-assisted discrimination on resource-constrained devices.

**Figure 11:**
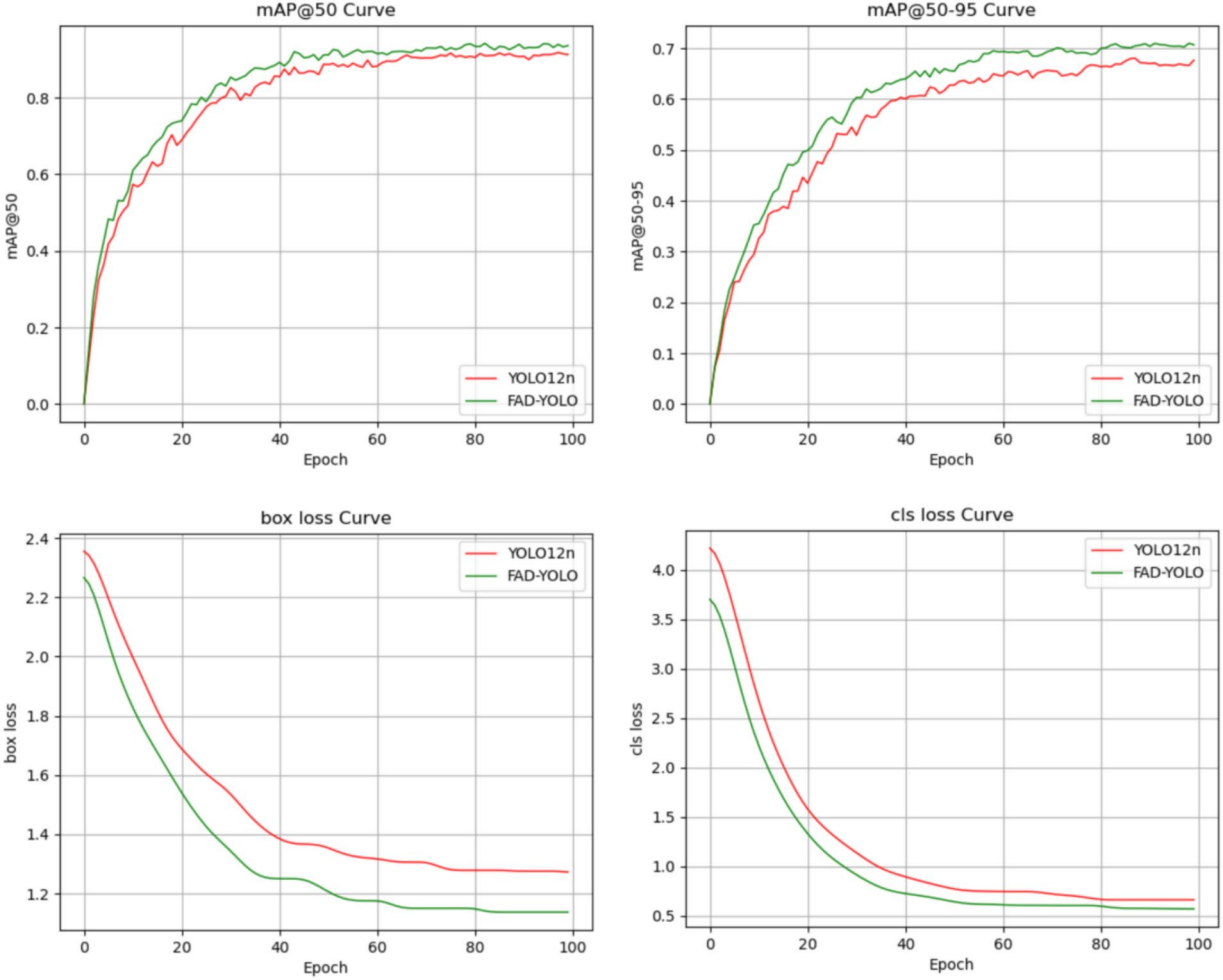
Training curves of mAP@50, mAP@50–95, box loss, and classification loss on fold-3 of the five-fold cross-validation.

Figure 11 presents the training curves of FAD-YOLO and the YOLO12n baseline on the validation set of fold-3, a representative fold from the five-fold cross-validation, including four metrics: mAP@50, mAP@50–95, box loss, and classification loss. Both models were trained for 100 epochs; the metrics of FAD-YOLO began to stabilize after approximately the 80th epoch, while those of YOLO12n stabilized after approximately the 85th epoch, indicating consistent convergence behavior between the two. Despite a substantial reduction in parameters and only a modest increase in computation relative to the baseline, FAD-YOLO outperformed the baseline on all metrics.

In summary, under the experimental conditions established in this study, FAD-YOLO achieves comparable or superior performance relative to all evaluated baselines across detection metrics, attaining a favorable balance among lightweight design, accuracy, and real-time inference.

### 4.6 Generalization assessment

To evaluate the generalization capability of FAD-YOLO, Dataset 2 was used as an external independent test set. Its substantial differences from Dataset 1 in acquisition workflow, patient composition, and scanner parameters provide a reasonably comprehensive simulation of the distribution shifts encountered in real clinical practice and enable an unbiased assessment of model performance on unseen data. FAD-YOLO was evaluated directly using the optimal weights trained on Dataset 1, without any fine-tuning, and the evaluation metrics were identical to those described above. Per-class and overall performance are reported in Table 5.

**Table 5:** Generalization assessment results on the external test set.

Class	P/%	R/%	mAP@50/%	mAP@50–95/%
AIS	89.8	89.3	90.4	66.8
MIA	91.6	91.2	93.0	69.6
All	90.7	90.2	91.7	68.2

As shown in Table 5, FAD-YOLO attained an overall performance of *p* = 90.7%, R = 90.2%, mAP@50 = 91.7%, and mAP@50–95 = 68.2% on the external test set. Compared with the internal test set (mAP@50 = 93.6%; mAP@50–95 = 70.8%), mAP decreased by 1.9–2.6 percentage points and P and R by approximately 3.1–3.2 percentage points, values that fall within a reasonable range of cross-dataset variability, indicating that the model maintains stable performance under different imaging conditions and confirming its cross-dataset generalization.

At the class level, MIA outperformed AIS across all metrics (MIA: *p* = 91.6%, R = 91.2%, mAP@50 = 93.0%, mAP@50–95 = 69.6%), consistent with its pathological characteristics: MIA lesions typically contain a small invasive component (≤5 mm) and most often appear as mixed GGNs with focal solid components, exhibiting discernible density contrast against the adjacent pulmonary parenchyma and providing more salient visual cues. AIS yielded slightly lower metrics (*p* = 89.8%, R = 89.3%, mAP@50 = 90.4%, mAP@50–95 = 66.8%) but remained at an acceptable overall level. The relatively lower performance for AIS is closely related to its inherent pathological and imaging characteristics: AIS almost invariably presents as a pure ground-glass opacity (pGGO) without solid components, exhibits weak density contrast with the surrounding parenchyma, and has diffuse boundaries, making it prone to false positives (misinterpretation of normal vessels or parenchymal patterns as AIS) and false negatives (missed detection of AIS with very faint density). Under heterogeneous data from multiple institutions, differences in scanner models, reconstruction algorithms, and scanning parameters further amplify this difficulty, as is clearly reflected in the more pronounced cross-dataset performance drop for AIS. Enhancing the robustness of pGGO-type AIS detection under heterogeneous data therefore constitutes an important direction for future research.

**Figure 12:**
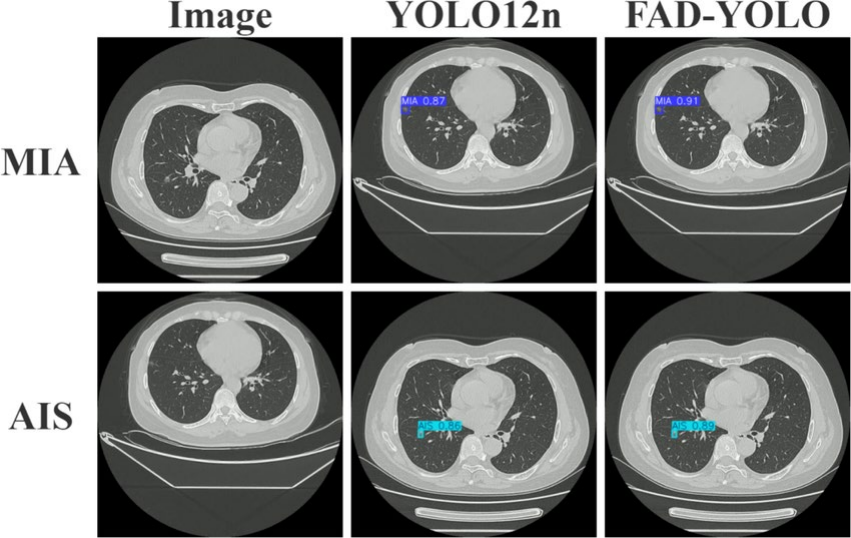
Comparison of detection results before and after model improvement on the external test set.

Figure 12 displays the visualized detection results of the model before and after improvement on the external test set. FAD-YOLO accurately localizes AIS and MIA lesions with closely fitted bounding boxes and maintains consistent detection quality across both classes, providing intuitive evidence of its cross-dataset generalization.

Overall, the external performance of FAD-YOLO is close to its internal performance, indicating that the learned representations are transferable rather than overfit to the training distribution and that the model has potential for deployment in diverse clinical scenarios.

### 4.7 Heatmap analysis

**Figure 13:**
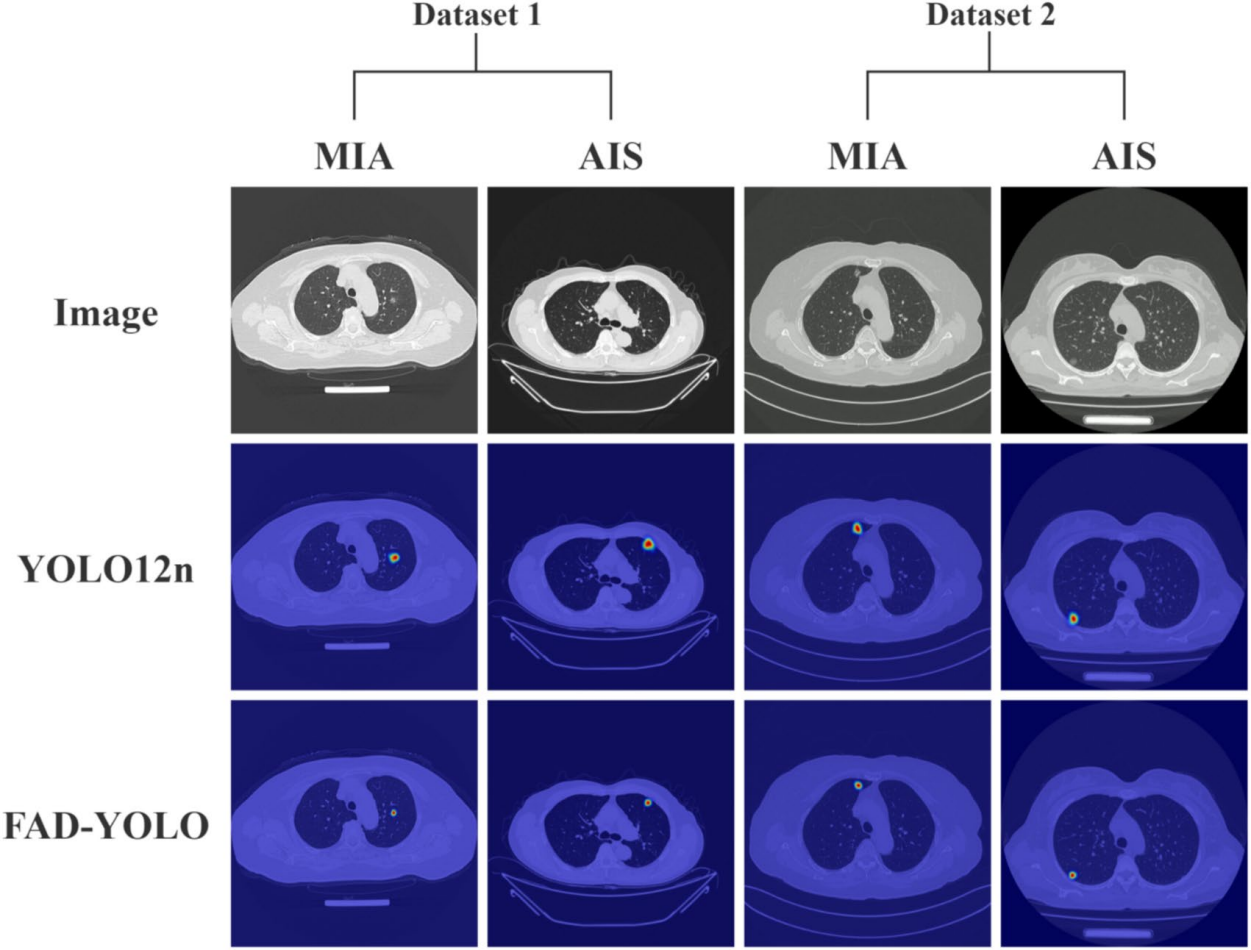
Attention heatmaps for AIS and MIA on Dataset 1 and Dataset 2.

To intuitively understand the decision mechanism of FAD-YOLO and its attention to lesion regions, Gradient-weighted Class Activation Mapping (Grad-CAM) was employed to generate attention heatmaps. The top 2% of detection boxes by confidence served as the back-propagation target; gradients were jointly computed from the classification logits and bounding-box regression and used to generate the class activation maps, in order to verify whether the model’s decisions rely on AIS/MIA-specific regions rather than on background noise or irrelevant anatomical structures. The target layers were chosen as the multi-scale fusion output layers of the detection head: the baseline model uses the A2C2f modules at layers 11, 14, and 17, whereas FAD-YOLO uses the corresponding multi-scale feature outputs of the substituted A2C2f-FRFN. In the heatmaps, red denotes high activation (regions of attention), and blue denotes low activation; heatmaps were generated separately on the internal (Dataset 1) and external (Dataset 2) test sets to compare attention patterns under in-distribution and out-of-distribution data. The results are shown in Figure 13.

In Figure 13 (top row: original CT; middle row: YOLO12n heatmap; bottom row: FAD-YOLO heatmap), the activations of FAD-YOLO are more precisely focused on the core and edges of the GGN, are more compactly distributed, and diffuse markedly less into the surrounding pulmonary parenchyma, vessels, and airways. For MIA, FAD-YOLO’s activations accurately cover the mixed ground-glass region and the focal solid component, exhibit substantially fewer false activations on adjacent normal textures, and produce relatively sharp responses at the solid/ground-glass density-transition boundary, indicating that the model effectively captures the intrinsic heterogeneity of MIA. For AIS, despite the inherent difficulty of localizing pure ground-glass lesions with low contrast and diffuse boundaries, FAD-YOLO’s responses still concentrate tightly on the lesion extent, with activation boundaries that conform more closely to the lesion contour than the diffuse activations of the baseline, reflecting improved discriminative capability under low-contrast and blurred-boundary conditions.

These improvements in activation patterns arise from the synergistic operation of the three modules: A2C2f-FRFN, through FRFN’s gated multiplicative interaction between an activation-carrying branch and a depthwise-refined modulating branch, selectively amplifies discriminative responses associated with subtle density differences and lesion edges in GGNs while suppressing background responses from vessels and parenchymal textures; DySample generates content-dependent offsets during upsampling, preserving the resolution of critical boundaries and density-transition regions throughout the neck pathway and providing a basis for fine-grained heatmap focus; and TADDH aligns the spatial focus of classification and localization through a shared Conv_GN stage, deformable sampling within the localization branch, and layer-attention reweighting, allowing activations to conform more closely to the true lesion contour while suppressing high responses at non-target locations.

Further comparison between the two datasets reveals that the activation patterns of FAD-YOLO remain highly consistent across the internal and external test sets, with attention stably concentrated on the lesion core and no apparent deviation or false activation attributable to distribution shift. This provides qualitative evidence of cross-dataset stability and offers an intuitive reference for clinicians to interpret AI-assisted diagnostic decisions.

## 5 Discussion

This study proposes FAD-YOLO to address several key limitations of YOLO12 in the discrimination of AIS and MIA on pulmonary CT. The contribution of this work lies in task-oriented engineering-level integration and domain adaptation: A2C2f-FRFN replaces the standard FFN within the A2C2f module with a feature-refinement feed-forward network that uses gated multiplicative interaction to selectively emphasize salient activations; DySample replaces static nearest-neighbor interpolation with content-aware learnable upsampling; and TADDH introduces a shared Conv_GN stage, deformable sampling within the localization branch, and layer-attention reweighting into the detection head. These three components are jointly designed to address the low contrast, blurred boundaries, and morphological variability of GGNs, forming a unified detection framework that balances high accuracy with computational efficiency under resource-constrained conditions.

The ablation study shows that the gains of all three modules on mAP@50–95 consistently exceed those on mAP@50, indicating that each module contributes more substantially under stringent localization criteria, precisely where general-purpose backbones tend to degrade most on low-contrast nodules, in line with the design intent of each component. A2C2f-FRFN couples gated multiplicative interaction with depthwise spatial refinement to selectively amplify discriminative activations, preserving fine-grained lesion textures that are otherwise smoothed by uniform channel-wise transformations. DySample converts upsampling from static replication into a content-aware geometric transformation, explicitly preserving GGN boundary sharpness and the continuity of internal density gradients. TADDH aligns the spatial focus of classification and regression through a shared Conv_GN stage and deformable sampling within the localization branch, and applies channel-wise dynamic weighting through layer-attention reweighting, suppressing redundant predictions caused by classification–localization misalignment. Unlike capacity-oriented strategies that pursue performance through deeper or wider networks, the modules proposed here prioritize efficient utilization of task-specific features, as evidenced by the simultaneous 18.7% reduction in parameters relative to the baseline, only a marginal 12.5% increase in computational cost, and a substantial accuracy gain. The Grad-CAM visualizations in Section 4.7 further corroborate this interpretation: FAD-YOLO’s activations conform to lesion contours markedly better than those of the baseline, particularly at low-contrast boundaries where the baseline frequently produces diffuse, off-target responses.

Comparisons with mainstream detectors further underscore the necessity of domain adaptation for medical imaging. Although the YOLO nano series performs well on general-purpose object-detection benchmarks, it exhibits a clear accuracy ceiling on fine-grained tasks such as AIS/MIA discrimination. Tajbakhsh et al. (32) systematically analyzed the challenges faced by deep CNNs in medical imaging, including domain shift, intra-class heterogeneity, and label scarcity, and emphasized the critical role of task-oriented architectural adjustments. Hosny et al. (33) likewise observed in their review of artificial intelligence in radiology that direct transfer of general-purpose architectures generally fails to meet the demands of clinical fine-grained discrimination and that deep adaptation to imaging modality and lesion characteristics is required. Our design follows these recommendations only in part: FAD-YOLO adapts the backbone, neck, and head to the density and boundary characteristics of GGNs and, consistent with this, achieves a higher mAP@50–95 than RT-DETR-R18 with substantially fewer parameters and reduced computation; however, the domain shift and label scarcity that these works emphasise are only partially mitigated by a single-centre training set, which is why the multi-centre validation discussed below remains necessary. Task-oriented fine-tuning is therefore best read as a contributing factor rather than a complete solution.

On the external independent test set, FAD-YOLO’s performance dropped by only 1.9–2.6 percentage points relative to the internal test, demonstrating favorable cross-dataset stability. AlBadawy et al. (34) reported in a multi-center study that differences in scanners, reconstruction algorithms, and imaging protocols often lead to noticeable performance degradation when models are deployed across institutions; the stable performance of the present study under heterogeneous data thus partially corroborates the robustness of the proposed adaptations to distribution shift. The slightly lower metrics for AIS than for MIA are closely related to its pathological characteristics: AIS almost invariably appears as a pure GGO without solid components, exhibits very low contrast against the pulmonary parenchyma, and has diffuse boundaries, making it diagnostically challenging even for experienced radiologists. Kim et al. (35) further confirmed that the low contrast and diffuse growth pattern of pure ground-glass nodules markedly increase the difficulty of cross-center recognition, which aligns with the more pronounced cross-dataset performance drop observed for AIS in this study. Future work may consider incorporating multi-phase contrast-enhanced CT or 3D thin-section voxel fusion to provide the model with richer discriminative information.

Several limitations of this study should be acknowledged. Varoquaux et al. (36) have noted that single-source dataset evaluation is prone to optimistic bias and that existing medical imaging AI studies often overestimate true generalization; the training data in this work originate from a single clinical center and may not fully capture multi-center imaging variability, while the single external dataset of 300 images provides only preliminary evidence of generalization. Moreover, this public external dataset does not report acquisition parameters such as scanner models, slice thickness, or reconstruction kernels, so a fully matched cross-dataset comparison of acquisition conditions was not possible; larger-scale, multi-center validation with documented acquisition parameters is therefore required before the generalization of FAD-YOLO can be regarded as robust. Second, this study covers only the discrimination between AIS and MIA and does not include other subtypes such as invasive adenocarcinoma or benign GGNs; future work may extend the task to a more complete pathological spectrum. Third, this study employs single-window 2D slice analysis with image-level annotations, and reports neither patient-level metrics nor 3D voxel segmentation; the LIDC-IDRI benchmark established by Armato et al. (37) provides a large-scale resource for pulmonary nodule research, and subsequent studies based on it have demonstrated the clear advantages of 3D voxel and multi-parametric modeling for nodule characterization, making both important directions for future extension. Regarding class balance, the AIS (1,058 images) and MIA (1,064 images) sample sizes in Dataset 1 are nearly identical, and the external test set contains 150 images per class, providing a fair basis for comparison and reducing potential bias from class imbalance. In addition, mapping the 12-bit CT data to an 8-bit grayscale range through a fixed lung window (W = 1,500 HU, L = −600 HU) discards HU information outside the window and quantizes the intensity range. The lung window was adopted because AIS and MIA manifest as ground-glass nodules whose discriminative density differences lie within this range, and 8-bit lung-window images are the standard input for the detectors compared here, ensuring a consistent pipeline; nevertheless, the attendant loss of fine HU precision may attenuate very subtle density differences, and exploiting the full 12-bit dynamic range or multi-window inputs is a worthwhile direction for future work. Finally, the present evaluation uses detection-oriented metrics; a dedicated diagnostic-accuracy analysis with a pre-specified operating point (including confusion matrices, sensitivity, specificity, and per-class AUROC), lesion- and patient-level aggregation, calibration of the output probabilities, and a size-stratified assessment of sub-centimeter nodules are important next steps that we plan to pursue on a larger, multi-centre cohort in future clinical-validation work.

From the perspective of clinical deployment, this study lacks systematic validation involving clinicians in the loop, and its real-world utility must therefore be assessed through prospective clinical trials. False positives may lead to unnecessary follow-up or surgical intervention, whereas false negatives may delay early intervention for AIS or MIA; both scenarios entail clinical risks and require comprehensive cost–benefit analysis. Deployment further involves PACS integration, regulatory approval, and robustness across heterogeneous scanning protocols at different institutions. The intended application of the proposed model is as a computer-aided detection tool that assists radiologists in image interpretation, with final diagnostic decisions remaining with clinicians within a human–AI collaborative workflow. We further acknowledge that this study includes neither a direct comparison with radiologist performance nor a calibration analysis of the model’s output probabilities (e.g., calibration curves or Brier scores); both are important for clinical decision support and, together with clinician-in-the-loop evaluation, should be addressed in future prospective studies.

In summary, FAD-YOLO achieves a comprehensive balance among detection accuracy, lightweight design, and generalization for AIS/MIA discrimination on pulmonary CT. Future work will focus on large-scale prospective multi-center clinical validation, multimodal fusion of multi-phase or 3D voxel data, and inference-time optimization tailored to clinical deployment.

## 6 Conclusion

This paper proposes FAD-YOLO, which integrates three targeted improvements, namely A2C2f-FRFN, DySample, and TADDH, to address three practical limitations of YOLO12 in AIS/MIA discrimination on pulmonary CT: namely, the inadequate characterization of subtle density and high-frequency edge cues by the standard feed-forward network, the disruption of lesion boundaries and density transitions caused by the neck’s nearest-neighbor upsampling, and the spatial misalignment between the classification and regression branches in the decoupled head. On the internal independent test set, the model achieved *p* = 93.8%, R = 93.4%, mAP@50 = 93.6%, and mAP@50–95 = 70.8%, with an 18.7% reduction in parameters and a slight rise in computational cost from 6.4 to 7.2 GFLOPs compared to the baseline; under identical experimental conditions, its performance is comparable to or better than that of all evaluated baselines. On the external independent test set, the model, evaluated without fine-tuning, achieved mAP@50 = 91.7% and mAP@50–95 = 68.2%, further corroborating its cross-dataset generalization. Collectively, these results indicate that FAD-YOLO may serve as a candidate for further prospective validation as an aid to radiologists in the automated discrimination of AIS and MIA on computationally constrained clinical devices.

## Data Availability

The datasets and code presented in this article are not readily available because the CT imaging data of Dataset 1 are subject to institutional ethics restrictions (approval no. YJS2025-147-02, under which informed consent was waived). De-identified data, together with the training and evaluation code, the trained model weights, the configuration files, and the patient/lesion split assignments, are available from the corresponding author on reasonable request, subject to approval by the institutional ethics committee. Dataset 2 is publicly available from the Mendeley Data repository (https://data.mendeley.com/datasets/h7b4ryrbzw).
